# Supplementary material for: Testing the influence of environmental heterogeneity on fish species richness in two biogeographic provinces
Source: PeerJ. 2015 Feb 12;3:e760. doi: 10.7717/peerj.760 (PMC4330905; doi:10.7717/peerj.760)
Supplement: Appendix S1 [file peerj-03-760-s001.pdf]

Appendix S1 *Summary of fish species sampled in the two biogeographic provinces.*

Table 1: Summary of fish species sampled in the two biogeographic provinces.

Order	***Family***	***Genus***	***Species***	**Mean abundance (catch per unit effort)**	**Mean fork length (cm)**
Acipenseriformes	*Acipenseridae*	*Acipenser*	*brevirostrum*	3.67	59.90
			oxyrinchus	2.50	27.81
	Polyodontidae	*Polyodon*	*spathula*	1.50	14.25
Anguilliformes	*Anguillidae*	*Anguilla*	*rostrata*	1.38	43.64
	Congridae	*Conger*	*oceanicus*	1.00	20.10
Atheriniformes	*Atherinopsidae*	*Membras*	*martinica*	12.33	6.69
		Menidia	*menidia*	2.50	6.72
Aulopiformes	*Synodontidae*	*Synodus*	*foetens*	1.90	15.92
Batrachoidiformes	*Batrachoididae*	*Opsanus*	*beta*	2.58	15.30
			pardus	1.50	16.52
			tau	4.05	19.29
		Porichthys	*plectrodon*	1.20	28.51
Beloniformes	*Belonidae*	*Strongylura*	*marina*	6.00	20.15
Carcharhiniformes	*Carcharhinidae*	*Carcharhinus*	*isodon*	1.00	38.00
			limbatus	1.00	39.20
			porosus	1.00	50.50
	Sphyrnidae	*Sphyrna*	*tiburo*	1.00	37.47
	Triakidae	*Mustelus*	*canis*	4.67	53.95
Clupeiformes	*Clupeidae*	*Alosa*	*aestivalis*	3.62	7.95
			pseudoharengus	6.38	7.35
			sapidissima	7.33	10.43
		Brevoortia	*gunteri*	35.17	7.99
			patronus	20.81	8.79
			smithi	1.00	12.20
			tyrannus	5.69	13.19
		Clupea	*harengus*	21.11	10.08
		Dorosoma	*cepedianum*	14.26	16.91
			petenense	11.85	10.37
		Etrumeus	*teres*	1.00	20.00
		Harengula	*jaguana*	2.47	9.70
		Opisthonema	*oglinum*	2.22	8.46
		Sardinella	*aurita*	3.00	13.07
	Engraulidae	*Anchoa*	*hepsetus*	8.66	6.81
			mitchilli	14.00	5.12
Cypriniformes	*Catostomidae*	*Carpiodes*	*cyprinus*	12.00	38.84
		Catostomus	*commersoni*	2.00	22.07
		Moxostoma	*macrolepidotum*	1.00	37.40
	Cyprinidae	*Carassius*	*auratus*	2.50	26.49
		Cyprinus	*carpio*	2.83	57.31
		Notemigonus	*crysoleucas*	2.00	14.00
		Notropis	*atherinoides*	10.00	9.71
			hudsonius	2.67	8.17
Cyprinodontiformes	*Fundulidae*	*Fundulus*	*grandis*	14.00	9.95
Elopiformes	*Elopidae*	*Elops*	*saurus*	1.14	21.20
Gadiformes	*Gadidae*	*Microgadus*	*tomcod*	10.30	9.41
		Pollachius	*virens*	1.00	13.10
	Lotidae	*Enchelyopus*	*cimbrius*	1.50	21.48

	Merlucciidae	*Merluccius*	*bilinearis*	2.00	20.98
	Phycidae	*Urophycis*	*chuss*	3.67	24.75
			regia	4.62	21.65
			tenuis	3.86	21.56
Gasterosteiformes	*Gasterosteidae*	*Gasterosteus*	*aculeatus*	1.00	7.80
Lampriformes	*Trachipteridae*	*Desmodema*	*polystictum*	1.00	16.95
Lepisosteiformes	*Lepisosteidae*	*Atractosteus*	*spatula*	1.00	48.00
		Lepisosteus	*oculatus*	1.00	47.00
			osseus	4.00	91.18
Lophiiformes	*Ogcocephalidae*	*Ogcocephalus*	*radiatus*	1.00	19.70
Mugiliformes	*Mugilidae*	*Mugil*	*cephalus*	2.89	17.53
			curema	1.57	12.21
Ophidiiformes	*Ophidiidae*	*Ophidion*	*marginatum*	1.50	16.85
Osmeriformes	*Osmeridae*	*Osmerus*	*mordax*	1.00	12.15
Perciformes	*Carangidae*	*Carangoides*	*ruber*	6.00	6.48
		Caranx	*crysos*	2.00	15.07
			hippos	3.09	8.92
			latus	2.00	8.91
		Chloroscombrus	*chrysurus*	25.50	7.31
		Decapterus	*punctatus*	7.00	13.21
		Hemicaranx	*amblyrhynchus*	2.67	12.01
		Selar	*crumenophthalmus*	7.00	9.40
		Selene	*setapinnis*	1.62	6.00
			vomer	2.14	5.60
		Trachinotus	*carolinus*	2.00	9.89
			falcatus	4.50	6.98
	Centrarchidae	*Lepomis*	*gibbosus*	8.14	11.13
			macrochirus	4.86	14.94
			microlophus	3.00	10.13
		Micropterus	*salmoides*	1.00	10.50
		Pomoxis	*annularis*	1.40	15.12
	Centropomidae	*Centropomus*	*undecimalis*	1.00	24.00
	Chaetodontidae	*Chaetodon*	*ocellatus*	2.00	4.85
	Ephippidae	*Chaetodipterus*	*faber*	3.13	7.47
	Gerreidae	*Eucinostomus*	*argenteus*	3.20	7.03
			gula	1.40	9.18
			lefroyi	6.50	7.64
	Gobiidae	*Gobioides*	*broussonneti*	1.00	19.60
		Gobiosoma	*bosc*	3.00	3.63
	Haemulidae	*Haemulon*	*plumieri*	9.50	8.96
		Orthopristis	*chrysoptera*	7.60	12.00
	Labridae	*Halichoeres*	*caudalis*	1.00	11.50
		Lachnolaimus	*maximus*	5.00	6.80
		Tautoga	*onitis*	10.60	17.06
		Tautogolabrus	*adspersus*	4.11	10.22
	Lutjanidae	*Lutjanus*	*griseus*	2.00	10.30
			synagris	2.67	8.16
	Moronidae	*Morone*	*americana*	70.75	13.94
			saxatilis	10.09	15.59
	Mullidae	*Upeneus*	*parvus*	1.00	8.60
	Percidae	*Etheostoma*	*olmstedi*	1.00	2.50
		Perca	*flavescens*	14.00	17.60
	Pholidae	*Pholis*	*gunnellus*	2.00	10.80
	Polynemidae	*Polydactylus*	*octonemus*	1.91	11.45
	Pomatomidae	*Pomatomus*	*saltatrix*	2.21	16.78
	Priacanthidae	*Priacanthus*	*arenatus*	1.00	10.60
	Rachycentridae	*Rachycentron*	*canadum*	2.00	21.03
	Scaridae	*Sparisoma*	*radians*	4.00	12.63

	Sciaenidae	*Aplodinotus*	*grunniens*	4.00	14.85
		Bairdiella	*chrysoura*	6.09	12.56
			sanctaeluciae	19.00	14.84
		Cynoscion	*arenarius*	12.43	11.20
			nebulosus	1.25	16.36
			nothus	1.83	12.23
			regalis	25.94	15.04
		Leiostomus	*xanthurus*	33.85	12.52
		Menticirrhus	*americanus*	1.25	20.14
			saxatilis	1.67	16.38
		Micropogonias	*undulatus*	21.83	14.04
		Odontoscion	*dentex*	1.00	15.80
		Pogonias	*cromis*	3.38	22.58
		Sciaenops	*ocellatus*	3.75	28.81
		Stellifer	*lanceolatus*	1.00	11.80
	Scombridae	*Scomberomorus*	*cavalla*	1.50	13.66
			maculatus	2.60	14.80
	Serranidae	*Centropristis*	*philadelphica*	2.00	12.71
			striata	3.43	14.22
		Diplectrum	*formosum*	1.00	12.57
		Mycteroperca	*bonaci*	1.00	14.50
			microlepis	1.00	23.20
		Serranus	*subligarius*	2.00	8.60
	Sparidae	*Archosargus*	*probatocephalus*	1.27	23.35
		Calamus	*arctifrons*	2.67	12.54
			leucosteus	1.00	17.00
		Diplodus	*holbrooki*	6.09	8.57
		Lagodon	*rhomboides*	20.05	10.12
		Stenotomus	*caprinus*	2.00	9.00
			chrysops	40.67	11.32
	Sphyraenidae	*Sphyraena*	*borealis*	4.00	10.40
	Stromateidae	*Peprilus*	*alepidotus*	5.65	6.31
			burti	3.00	7.16
			triacanthus	21.51	8.27
	Trichiuridae	*Trichiurus*	*lepturus*	2.06	32.90
	Uranoscopidae	*Astroscopus*	*guttatus*	1.00	14.15
Pleuronectiformes	*Achiridae*	*Achirus*	*lineatus*	2.00	8.65
		Gymnachirus	*texae*	1.00	7.50
		Trinectes	*maculatus*	17.44	9.93
	Cynoglossidae	*Symphurus*	*civitatium*	1.00	12.40
			plagiusa	6.41	13.61
	Paralichthyidae	*Ancylopsetta*	*ommata*	1.00	18.40
		Citharichthys	*macrops*	2.00	11.25
			spilopterus	2.71	8.48
		Etropus	*crossotus*	3.00	12.50
			microstomus	2.40	10.63
			sp	1.00	10.20
		Hippoglossina	*oblonga*	6.20	22.52
		Paralichthys	*albigutta*	1.67	25.47
			dentatus	3.15	26.34
			lethostigma	2.02	17.51
		Syacium	*gunteri*	1.00	10.20
	Pleuronectidae	*Pseudopleuronectes*	*americanus*	10.04	15.94
	Scophthalmidae	*Scophthalmus*	*aquosus*	6.29	15.69
Rajiformes	*Dasyatidae*	*Dasyatis*	*americana*	7.50	37.39
			centroura	1.00	77.00
			sabina	3.53	25.37
			say	2.00	42.58

	Gymnuridae	*Gymnura*	*altavela*	1.00	47.50
			micrura	2.50	51.50
	Myliobatidae	*Myliobatis*	*freminvillei*	1.33	45.33
		Rhinoptera	*bonasus*	1.33	35.75
	Rajidae	*Leucoraja*	*erinacea*	5.45	40.62
			ocellata	1.50	36.27
		Raja	*eglanteria*	3.25	54.74
Scorpaeniformes	*Cottidae*	*Myoxocephalus*	*aenaeus*	32.50	10.37
			octodecemspinosus	1.00	14.05
	Triglidae	*Prionotus*	*alatus*	1.00	11.95
			carolinus	5.27	13.23
			evolans	2.22	13.70
			roseus	4.00	10.95
			rubio	1.00	12.40
			tribulus	2.12	8.87
Siluriformes	*Ariidae*	*Ariopsis*	*felis*	7.43	19.21
		Bagre	*marinus*	15.79	11.22
	Ictaluridae	*Ameiurus*	*catus*	8.92	19.92
			natalis	5.00	22.44
			nebulosus	10.00	20.76
		Ictalurus	*furcatus*	45.82	17.56
			punctatus	15.46	21.34
Syngnathiformes	*Syngnathidae*	*Hippocampus*	*erectus*	1.17	11.20
		Syngnathus	*floridae*	1.00	16.50
			fuscus	1.00	20.80
			louisianae	3.67	13.36
			scovelli	1.00	20.00
Tetraodontiformes	*Diodontidae*	*Chilomycterus*	*reticulatus*	5.25	12.03
			schoepfi	1.80	16.05
		Diodon	*hystrix*	2.00	16.40
	Monacanthidae	*Aluterus*	*schoepfi*	1.00	16.03
			scriptus	3.33	17.26
		Stephanolepis	*hispidus*	4.75	9.91
			setifer	4.00	6.05
	Ostraciidae	*Acanthostracion*	*quadricornis*	1.56	15.02
	Tetraodontidae	*Canthigaster*	*rostrata*	1.00	8.00
		Lagocephalus	*laevigatus*	1.00	18.20
		Sphoeroides	*maculatus*	2.24	12.69
			nephelus	16.00	3.89
			parvus	4.13	6.29
