# Supplementary material for: Testing the influence of environmental heterogeneity on fish species richness in two biogeographic provinces
Source: PeerJ. 2015 Feb 12;3:e760. doi: 10.7717/peerj.760 (PMC4330905; doi:10.7717/peerj.760)
Supplement: Appendix S2 [file peerj-03-760-s002.pdf]

Appendix S2 *Simulation details and parametrization.*

Spatio-temporal Moran's I decomposition

The Moran's I_{global} statistic (equation 1) was computed by pooling all observations in each biogeographic provinces. To ensure that the measured local I_{global} reflected the spatial heterogeneity of environmental conditions rather than the temporal variability of environmental conditions, we decomposed I_{global} (equation 1) into its spatial and temporal components using equation 2.

$$I_{global} = (n-1)\frac{x_i - \bar{X}}{\sum\limits_{i=1}^{n}(x_i - \bar{X})^2}\sum_{j=1}^{n} w_{ij}(x_j - \bar{X}) \tag{1}$$

$$I_{temporal} = I_{global} - I_{spatial} \tag{2}$$

Where $I_{temporal}$, I_{global} and $I_{spatial}$ are respectively the *temporal*, *spatio-temporal* and *spatial* Moran's I. I_{global} was computed on data pooled at the level of biogeographic provinces whereas $I_{spatial}$ was computed by year for each biogeographic province. The results of the decomposition are presented in figure 1.

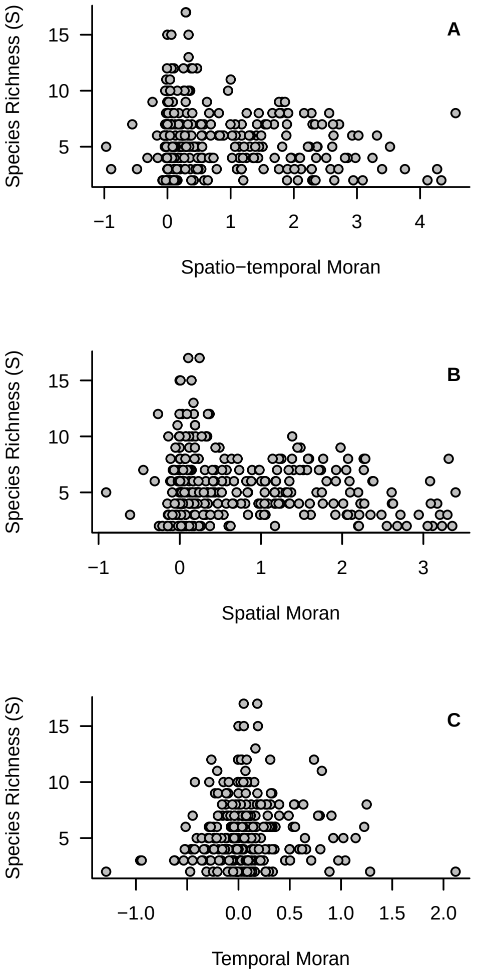

Figure 1: Results of the Moran's I decomposition. **(A)** Spatio-temporal data (used in this study), **(B)** Spatial Moran's I and **(C)** Temporal Moran's I.

Distribution ranges

To determine the spatial distribution range of each species (ellipse size), we used a long-tail distribution yielding small ranges for most species and large ranges for few species (Figure 2). The minimum and maximum distribution range thresholds were based on the observed regional distribution of species ($r_{min} \leq r \leq r_{max}$ with $r_{min} = 10$ km and $r_{min} = 1000$ km).

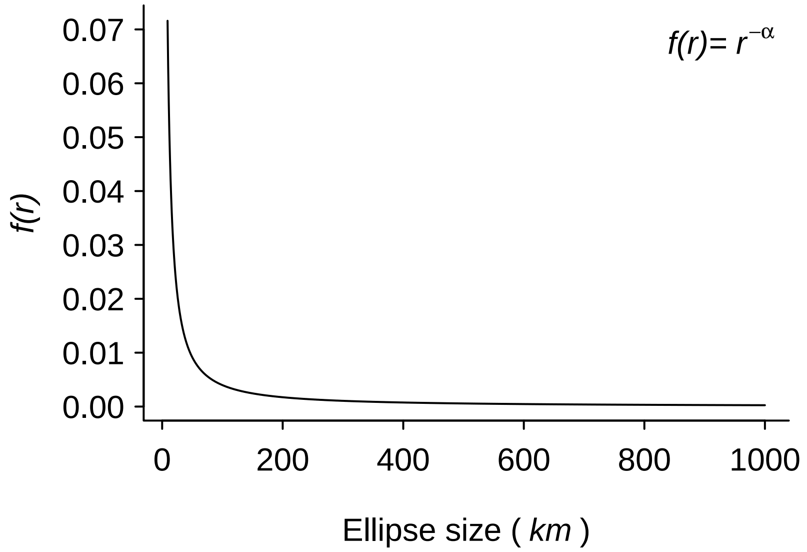

Figure 2: Power function used to randomly pick the distribution range r for species placed on the surface grid.

Examples of the regional distribution for four species found in the Louisianian biogeographic province.

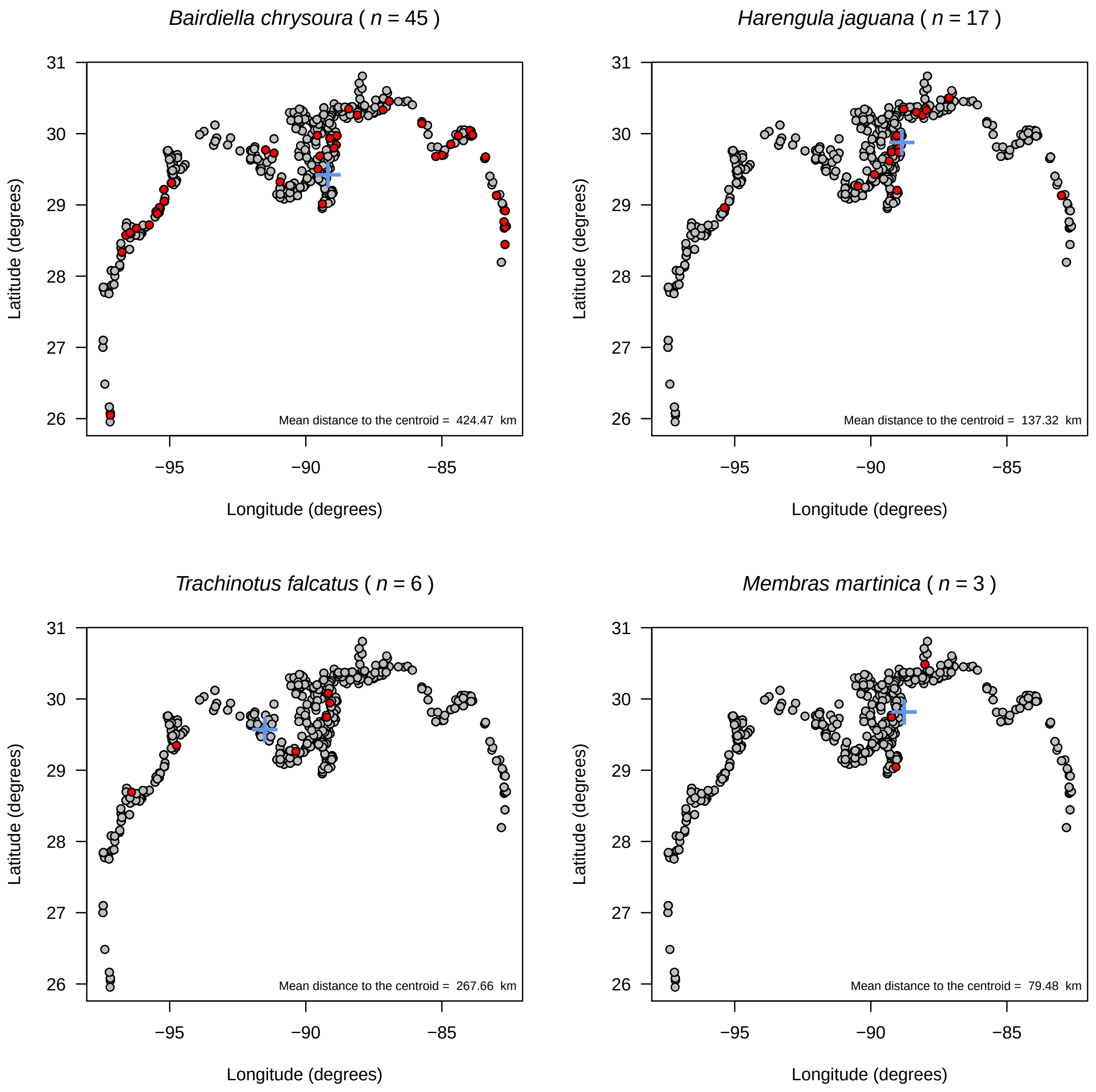

Figure 3: Examples of the regional distribution for four species found in the Louisianan biogeographic province. The red dots show the locations where each species was found whereas as the blue crosses are the centroids of these locations. The average distances to centroids for species found in Louisianan and Virginian biogeographic provinces are 134.4 km and 85.1 km respectively.

Example of connectivity graph used to identify neighbours of region points by Euclidean distance.

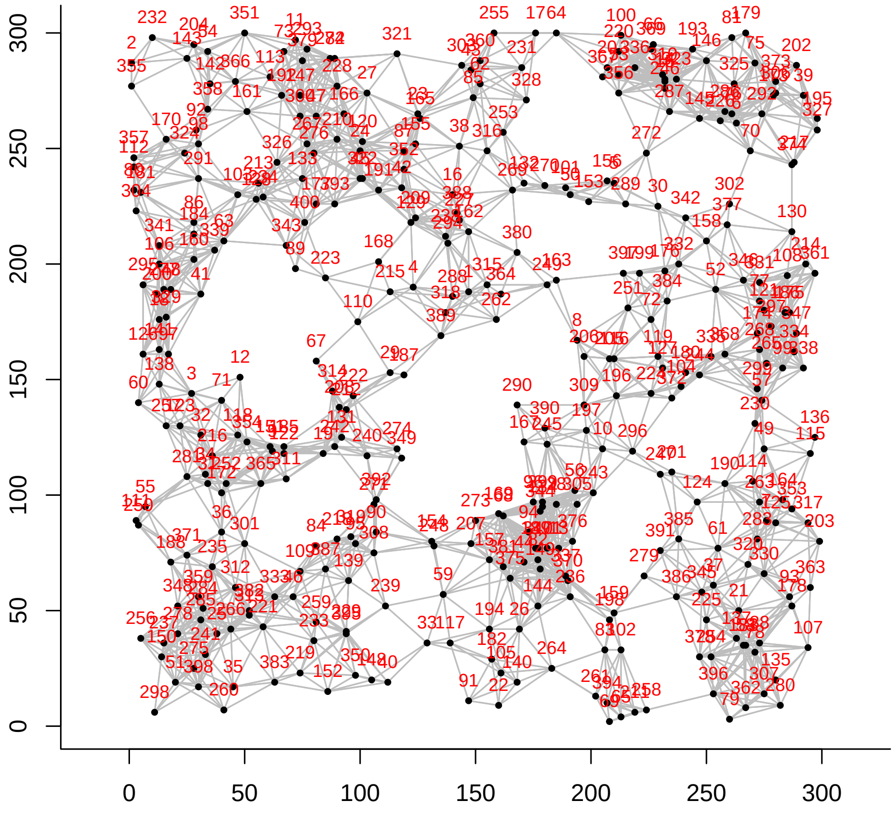

Figure 4: Connectivity graph identifying neighbours of region points falling under the 10% maximum range (see Methods for detailed information).
